# Targeting MutT Homolog 1 (MTH1) for Breast Cancer Suppression by Using a Novel MTH1 Inhibitor MA−24 with Tumor-Selective Toxicity

**DOI:** 10.3390/ph17030291

**Published:** 2024-02-23

**Authors:** Nannan Kang, Jun Ma, Yuling Hu, Rongrong Di, Lei Wang, Xuanling Zhang, Yisheng Lai, Yu Liu

**Affiliations:** 1 School of Life Science & Technology, China Pharmaceutical University, Nanjing 211198, China; kangnannan@cpu.edu.cn (N.K.); huyuling1234@163.com (Y.H.); dirongcpu@163.com (R.D.); 3322031117@cpu.edu.cn (X.Z.); 2 Center of Drug Discovery, China Pharmaceutical University, Nanjing 210009, China; majun_cpu@163.com (J.M.); wanglei_0922@163.com (L.W.)

**Keywords:** breast cancer, MutT homolog 1(MTH1), antitumor activity, oxidized nucleotide, MA−24

## Abstract

Background: Breast cancer is a commonly diagnosed cancer worldwide. Human MutT homolog 1 (MTH1) is found to be elevated in breast tumors and cancer cells need MTH1 for survival. Pharmacological inhibition of MTH1 may be potentially beneficial in the treatment of breast cancer. Methods: MA−24 was screened by malachite green colorimetric assay for MTH1 inhibitors and the kinetic characteristics of MA−24 were assessed. The features of MA−24’s binding with MTH1 were ascertained through molecular docking, and the cytotoxic activity of MA−24 was validated in vitro and in vivo. Target engagement assays, comet assay, and Western blot confirmed the intracellular target and mechanism of MA−24. Results: MA−24 shows potent antitumor bioactivity both in vitro and in vivo. MA−24 competitively inhibited the MTH1 and further induced DNA strand breaks, leading to increased apoptosis of cancer cells depending on the upregulation of the cleaved-caspase 3–cleaved-PARP axis. In particular, MA−24 exhibited a powerful efficacy and safety in vivo (tumor growth inhibition rate: 61.8%). Conclusions: MA−24 possesses a broad spectrum of breast cancer cytotoxicity and offered valuable insights for overcoming the challenges of chemotherapy-related toxicity, which holds great potential for the further development MA−24 as an anti-cancer drug.

## 1. Introduction

Breast cancer ranks as the top cancer type in terms of incidence and is the fifth leading cause of cancer death [1]. Although conventional chemotherapeutic agents exhibit significant inhibitory effects on breast cancer, they also nonspecifically damage normal cells, causing harm to the human body. Additionally, the development of drug resistance limits their therapeutic efficacy [2]. Targeted therapies, while demonstrating a remarkable effectiveness for specific subsets of breast cancer patients, are not universally applicable due to their limited specificity [3]. Targeting the commonly observed abnormal phenotypes in cancer cells offers a potential solution for overcoming these therapeutic limitations and serves as a promising avenue for comprehensive cancer treatment.

Reactive oxygen species (ROS), as byproducts of oxidative phosphorylation, exert dual effects on cancer cells. On the one hand, they mediate signaling pathways that promote cancer cell survival, proliferation, and metastasis. On the other hand, excessive ROS production triggers tumor suppression through the induction of DNA damage [4]. Carcinogen-induced cancer cells characterized by rapid proliferation and metabolic abnormalities commonly exhibit elevated levels of ROS [5]. Increased ROS can attack macromolecules within cells, leading to various types of oxidative damage. Among these, nucleic acid damage can alter or disrupt genetic information within the genome, posing significant risks to cancer cells, including programmed cell death. However, normal cells are not fatally harmed by normal levels of ROS. Hence, cancer cells typically upregulate several DNA damage repair proteins, including human MutT homolog 1 (MTH1), to maintain genome integrity [6]. 

To overcome the inhibitory effects of high levels of ROS, cancer cells activate adaptive mechanisms. Research suggests that cancer cells rely heavily on the base excision repair mechanism mediated by MTH1 to prevent the misincorporation of harmful oxidized nucleotides into the genome and thereby reduce genotoxicity [7]. As a nudix phosphohydrolase, MTH1 catalyzes the hydrolysis of oxidized nucleoside triphosphates, including 8-oxo-7,8-dihydro-2′-deoxyguanosine triphosphate (8-oxo-dGTP), 2-hydroxyadenosine triphosphate (2-OH-dATP), and N6-methyl-dATP, into their respective monophosphates, thereby preventing the incorporation of these oxidized deoxyribonucleoside triphosphates into DNA during replication and minimizing the resulting DNA instability [8,9].

This function of MTH1 ultimately protects cells from ROS-induced damage [10,11]. Compared to normal cells, MTH1 is overexpressed in many cancers, including breast cancer, brain tumors, lung cancer, gastric cancer, renal cancer, and colorectal cancer arising from ulcerative colitis [8,12,13]. In particular, MTH1 is found to be overexpressed in distinct subtypes of breast cancer, independent of the molecular and clinicopathological characteristics of the tumor [14]. This difference in expression is closely associated with the levels of ROS. Cancer cells with higher ROS levels rely more on MTH1 for cell survival due to intensified oxidative attacks, leading to increased MTH1 expression [10]. Although research has shown that a small amount of normal cells also express MTH1 and play a beneficial role, MTH1 can protect platelet mitochondria from oxidative damage and regulate platelet function and thrombus formation [15]. In neurodegenerative diseases, MTH1 plays a neuroprotective role by minimizing the accumulation of its substrate, 8-oxoguanine (8-oxoG), in the nuclear and mitochondrial genomes of patients with Alzheimer’s disease. This inhibition of glial cell proliferation and neuronal loss highlights MTH1’s role in preserving neuronal integrity [16]. But more importantly, MTH1 plays a crucial role in countering cell death induced by oxidative and reductive abnormalities commonly present in cancer cells. Additionally, high MTH1 expression correlates significantly with poor prognosis in various malignancies [12,17,18]. Therefore, targeting MTH1 to enhance the intracellular accumulation of oxidized nucleotides and thereby induce DNA damage can be considered a novel strategy for eradicating breast cancer. These findings provide new research directions for overcoming the challenges within breast cancer treatment.

The inhibition of MTH1 can exert anticancer effects by preserving normal cell viability and exhibits broad-spectrum anticancer activity; TH287 and TH588 are the first small-molecule inhibitors targeting MTH1, and their potent anticancer effects have sparked interest in MTH1 inhibitors among researchers [8]. As a result, various types of MTH1 inhibitors have been developed, primarily classified as 2-aminopyrimidine derivatives [19,20], (S)-crizotinib or pyrazoloquinoline derivatives [21], natural extracts [22,23], nucleoside analogs [24], Kettle’s three classes of inhibitors [25], and nanoparticles [26]. All of these demonstrate a significant inhibitory effect on MTH1. In the present work, we report a novel MTH1 inhibitor, MA−24, which exhibits potent tumor-specific anti-breast cancer activity both in vitro and in vivo. These findings provide insight and a candidate compound for the development of new therapies and drugs targeting the common abnormal phenotypes in breast cancer cells.

## 2. Results

### 2.1. Identification of Promising MTH1 Inhibitors

**Figure 1:**
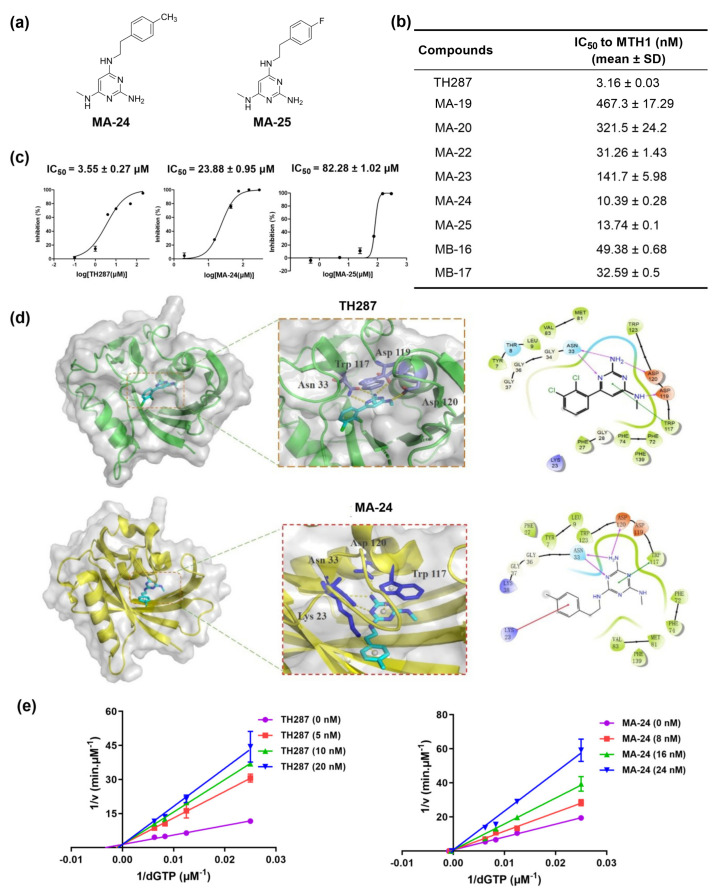
In vitro screening, protein-inhibitor molecular docking, and enzymatic characterization of MTH1 inhibitors. (**a**) Chemical structures of MA−24 and MA−25. (**b**) IC_50_ values (mean ± SD) of MA−19, MA−20, MA−22, MA−23, MA−24, MA−25, MA−16, and MB−17 against MTH1 enzyme determined by malachite green staining assay. (**c**) IC_50_ values (mean ± SD) of TH287, MA−24 and MA−25 against MCF−7 cell proliferation determined by MTT assay. (**d**) Binding mode of TH287 and MA−24 with MTH1 predicted by molecular docking using Schrödinger2009. The π−π stacking interactions are represented by cyan dash lines, π−cation interactions are represented by blue dash lines, and hydrogen-bonding interactions are represented by yellowish dash lines. (**e**) Kinetic analysis of TH287 and MA−24 inhibition of MTH1.

In order to screen for novel MTH1 inhibitors within the nine compounds we obtained (Figure S1), we constructed an enzymatic assay to determine the inhibitory activity of the target compounds against MTH1. All target compounds showed significant inhibitory activity against MTH1 except for MA−21, which did not have a determined IC_50_ value even at an extremely high dose (500 nM). Among them, compounds MA−24 and MA−25 displayed the best inhibitory activity with IC_50_ values of 10.39 ± 0.28 nM and 13.74 ± 0.1 nM, respectively, confirming a direct effect of MA−24 and MA−25 on MTH1 catalytic activity (Figure S1 and Figure 1a,b).

Next, we further evaluated the anti-proliferative potential of these compounds on breast cancer cells using the MTT assay. Initially, we determined the inhibitory effects of the compounds against MCF−7 cells at 200 μM. The results revealed that MA−24 and MA−25 exhibited stronger antiproliferative activity on MCF−7 cells than the other compounds (Table 1). Subsequently, different concentrations of MA−24, MA−25, and TH287, used as a positive control due to its structural similarity to MA−24 and MA−25 among all reported MTH1 inhibitors, were further used to determine their IC_50_ values against MCF−7 cells. Figure 1c demonstrates that MA−24 possesses a stronger ability to inhibit proliferation compared to MA−25, with IC_50_ values of 23.88 ± 0.95 μM and 82.28 ± 1.02 μM, respectively, while the IC_50_ value of TH287 is 3.55 ± 0.27 μM. Consequently, we selected MA−24 as the most promising compound for further activity exploration.

To elucidate the binding modes of MA−24 with the amino acids in the MTH1 peptide chain, molecular docking was performed using Schrödinger2009. The analysis indicated that MA−24 occupies the substrate binding site of MTH1 and forms critical interactions with nearby residues, similar to TH287. Briefly, the 1,3,5-triazine ring of MA−24 forms π-π stacking interactions with Trp117. Moreover, the nitrogen atom and the primary amine group of the 1,3,5-triazine core form three hydrogen bonds with Asn33 and Asp120, respectively. In addition, the distal benzene ring of MA−24 interacts with the backbone of Lys23 through additional carbonyl-π interactions (Figure 1d). These interactions contribute to the high affinity of MA−24 with the MTH1 protein.

In order to gain further insights into the type of inhibition MA−24 exhibits on MTH1, we conducted enzymatic kinetic exploration experiments. The reciprocal plot displayed in Figure 1e exhibits a consistent 1/Vmax regardless of the concentration variations of MA−24 or TH287. This observation suggests that MA−24 and TH287 exhibit a competitive inhibition type on MTH1 with respect to dGTP.

### 2.2. MA−24 Reduces Cell Survival and Induces Cell Apoptosis

**Figure 2:**
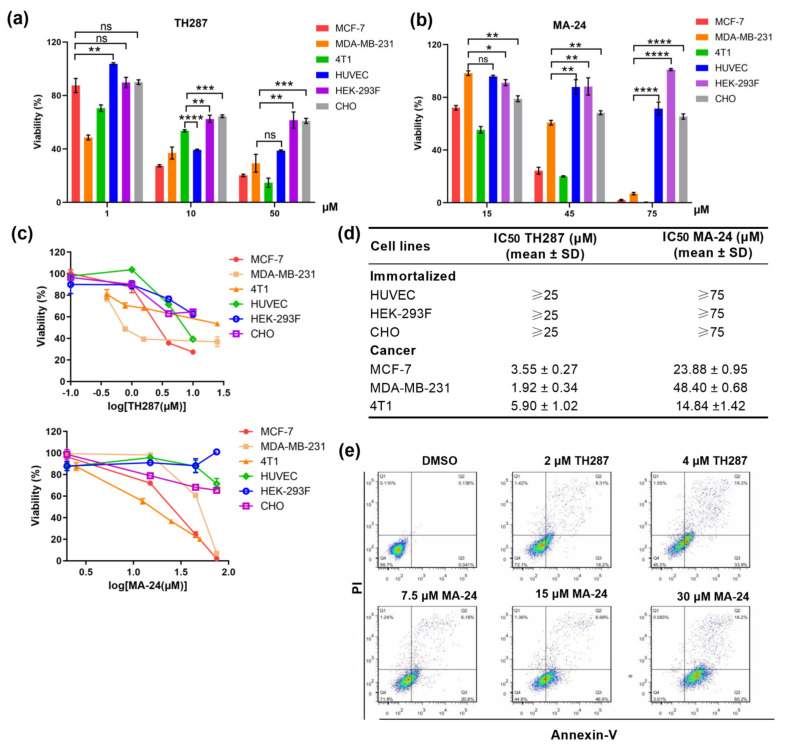
MTH1 inhibitor MA−24 reducing breast cancer cell viability and inducing apoptosis of MCF−7 cells. (**a**–**c**) Viability determined in breast cancer cell lines or immortalized cells after exposure to increasing concentrations of MTH1 inhibitors. *, *p* < 0.05, **, *p* < 0.01, ***, *p* < 0.001, ****, *p* < 0.0001, ns, no significant difference, *t*-test. (**d**) Determination of the IC_50_ values (mean ± SD) of TH287 and MA−24 on normal cells’ and tumor cells’ proliferation using the MTT assay. (**e**) Induction of cell apoptosis in MCF−7 cells by TH287 and MA−24 evaluated using flow cytometry.

Given the significant inhibitory activity of MA−24 on MTH1 protein and MCF−7 breast cancer cells, we further examined its anti-proliferative effects on other breast cancer cell lines and normal cell lines. As shown in Figure 2a–d, MA−24 was able to selectively and effectively suppress the proliferation of breast cancer cell lines, while it exerted a weaker toxicity towards several immortalized cells, which is similar to TH287. This is in agreement with normally non-essential MTH1 being of crucial importance to cancer cells [27]. In addition, we further observed a considerably increased proportion of Annexin V-positive apoptotic cells after MA−24 treatment in a dose-dependent manner (Figure 2e). These findings indicate that MA−24 exhibits selective cytotoxicity against tumor cell lines in vitro, while causing minimal damage to normal cells.

### 2.3. MTH1 as the Target of MA−24 Is a Major Determinant for the Survival of Cancer Cells

**Figure 3:**
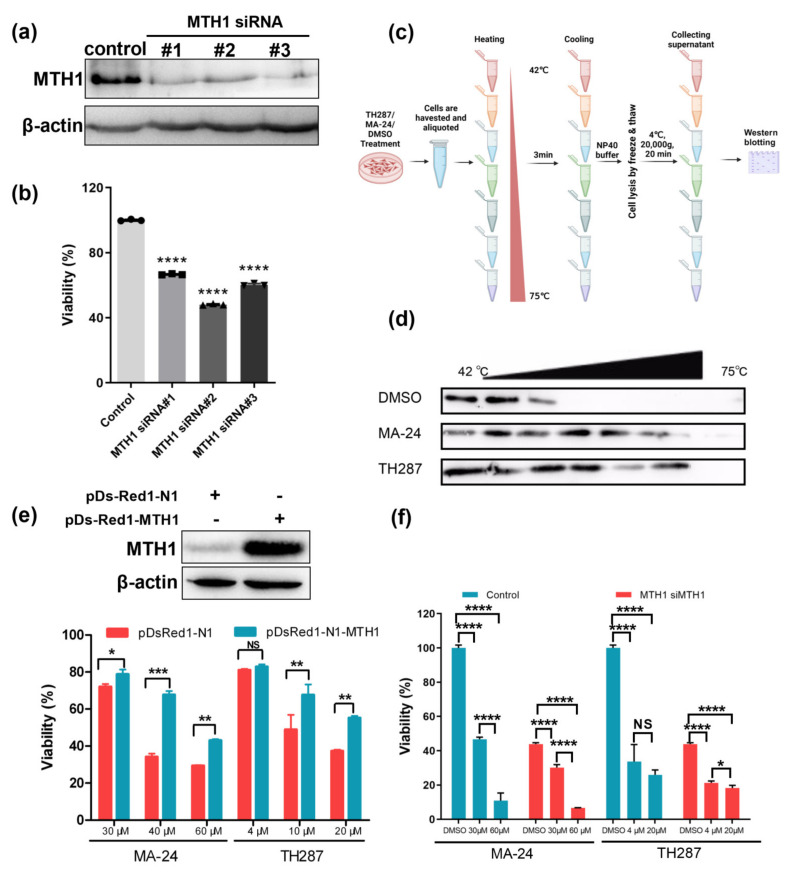
MTH1 as the target of MA−24 is a major determinant for the survival of cancer cells. (**a**) Western blot analysis demonstrating MTH1 silencing using siRNA. (**b**) MTT assay evaluating the impact of MTH1 silencing on the proliferation of MCF−7 cells. ****, *p* < 0.0001, *t*-test. (**c**) Schematic representation of the cellular thermal shift assay (CETSA) procedure, which is created using the BioRender website, (https://www.biorender.com/, accessed on 27 December 2023). (**d**) CETSA results showing that both MA−24 and TH287 bind to MTH1 and increase the thermal stability of the MTH1 protein. (**e**) Overexpression of MTH1 using pDs-Red1-MTH1 plasmid reverses the inhibitory effect of MTH1 inhibitors on the proliferation of MCF−7 cells. *, *p* < 0.05; **, *p* < 0.01; ***, *p* < 0.001, ns, no significant difference; *t*-test. (**f**) Under siRNA treatment, the inhibition of MTH1 intensifies the suppression of cellular viability. *, *p* < 0.05; ****, *p* < 0.0001, *t*-test.

The development of MTH1 inhibitors is primarily limited by two controversial issues: the non-essential role of MTH1 in cancer cells and the off-target effects of MTH1 inhibitors [25]. Hereon, we investigate them one by one with MCF−7 cells as models. To describe the relationship between MTH1 and breast cancer cell viability, we utilized three siRNAs which would lead to a decrease in the MTH1 protein (Figure 3a), and ultimately to the notable suppression of MCF−7 cells’ survival (Figure 3b). Collectively, these results demonstrate that MTH1 dominates the survivability of MCF−7 cells. Additionally, target engagement of MTH1 inhibitors was validated by cellular thermal shift assay (CETSA) (Figure 3c). The observation that MTH1 engaged by MA−24 shows prominent thermal stabilization is suggestive of it being target of MA−24, in concert with the biophysical principle of the ligand-induced thermal stabilization of target proteins (Figure 3d). More importantly, MTH1 status does influence the cytotoxicity induced by MA−24 or TH287, and the overexpression of MTH1 reversed the reduced survival of MCF−7 cells exposed to MTH1 inhibitors (Figure 3e). Nonetheless, MTH1 knockdown MCF−7 cells’ viability was also altered after treatment with the small molecule compounds MA−24 or TH287, which is potentially attributed to the incomplete deletion of MTH1 protein or the unknown off-target effects (Figure 3f). Overall, MTH1 is identified as the primary target after MA−24 treatment of viable cells and plays a critical role in cell survival.

### 2.4. MA−24 Causes Breast Cancer Cell Death Referring to Multiple Cellular Signaling Pathways

**Figure 4:**
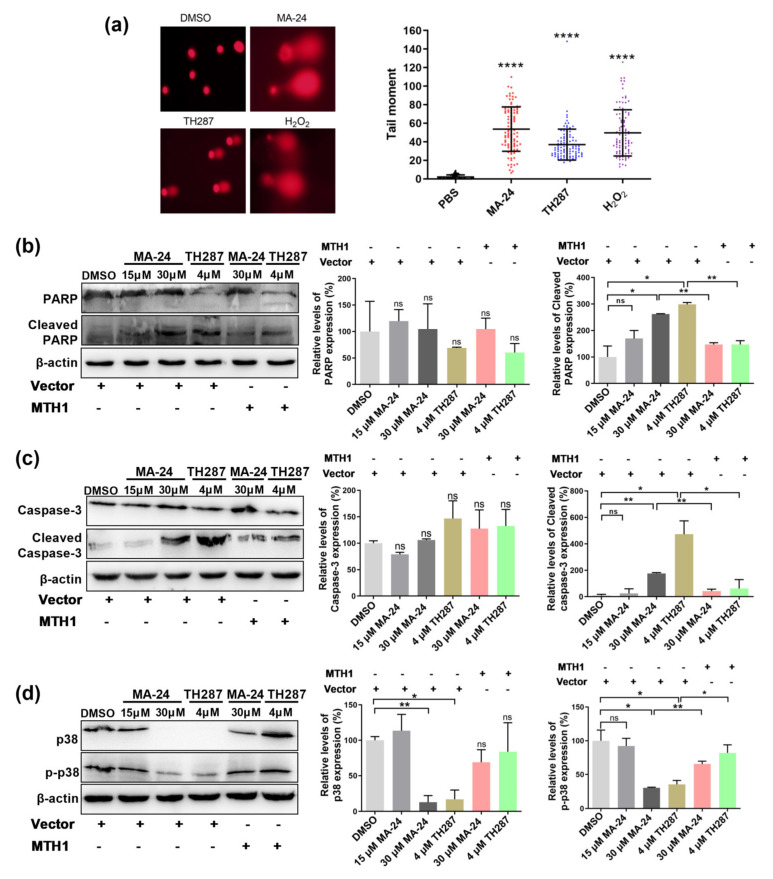
MA−24 promotes breast cancer cell death through multiple pathways. (**a**) Representative images and statistical results of comet assay in MCF−7 cells treated with DMSO (negative control), H_2_O_2_ (positive control), TH287, and MA−24. (Magnification: 20×). ****, *p* < 0.0001, *t*-test. (**b**) MA−24 significantly increases the expression of apoptotic marker protein cleaved-PARP in a dose-dependent manner, which can be reversed by MTH1 overexpression. *, *p* < 0.05, **, *p* < 0.01, ns, no significant difference, *t*-test. (**c**) MA−24 significantly enhances the expression of cleaved caspase-3, while MTH1 overexpression elevates the cleaved-caspase 3 levels induced by MA−24 and TH287. *, *p* < 0.05; **, *p* < 0.01. ns, no significant difference, *t*-test. (**d**) High concentrations of MA−24 and TH287 significantly reduce the expression of p38, and increasing MTH1 expression partially restores the decreased levels of p38 protein. MA−24 significantly inhibits the expression of p-p38 protein, and this reduction can be reversed by MTH1 overexpression. *, *p* < 0.05; **, *p* < 0.01, ns, no significant difference, *t*-test.

Since MTH1 inhibitors are thought to kill cancer cells by inducing DNA strand lesions, as a result of the cumulative 8-oxo-guanine (8-oxodG) and subsequent activation of base-excision repair (BER) [8]. Using an alkaline comet assay, we demonstrated that MA−24 significantly increased DNA strand breaks in MCF−7 cells, in line with the action of TH287 (Figure 4a). Because the expression of apoptosis-correlated proteins is known to be regulated by MTH1 inhibitors, we detected changes in the expression level of caspase 3, PARP, and their activated forms in MCF−7 cells after exposure to MA−24. In line with expectations, upregulation of cleaved-caspase 3 and cleaved PARP was observed while treated with 30 μM MA−24, which could be restored by the increase in the MTH1 protein (Figure 4b,c). While further confirming the mechanism of how MA−24 and TH287 induce cell apoptosis, we focused on the p38 MAPK pathway which is implicated in the survival of breast carcinoma cells. As is shown in Figure 4d, a decrease in p38 was measured upon treatment with small molecule inhibitors and overexpressing MTH1 would rescue the variation of p38 induced by MA−24. These results indicate that MA−24 induces DNA strand breaks, leading to the increased expression of cleaved-caspase 3 and PARP, and ultimately resulting in the apoptosis of MCF−7 cells.

### 2.5. MA−24 Inhibited Tumor Growth and Lung Metastasis In Vivo

**Figure 5:**
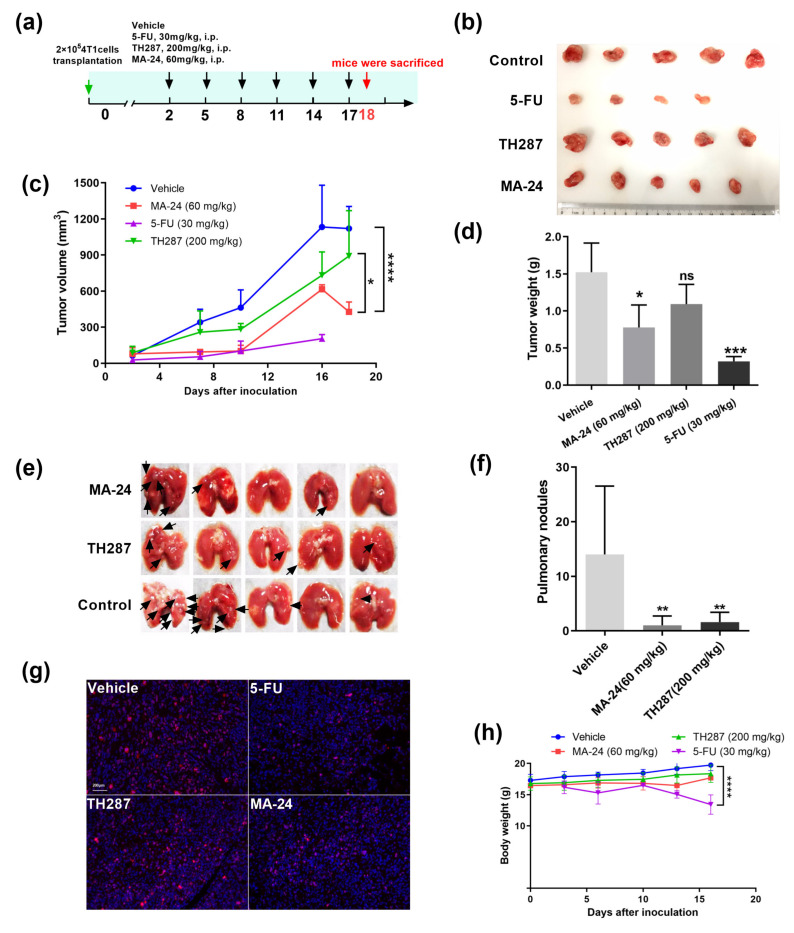
MA−24 exhibits superior tumor-inhibiting and lung metastasis-inhibiting abilities compared to the TH287 in vivo. (**a**) Experimental design for animal studies. (**b**) Tumor images from mice treated with vehicle control, 5-FU, TH287, and MA−24 after 18 days according to the experimental design. (**c**) Tumor growth curves in mice treated with vehicle control, 5-FU, TH287, and MA−24. Tumor volume was calculated using the formula V = D1 (long axis) × D2^2^ (short axis) × 0.52. *, *p* < 0.05; ****, *p* < 0.0001. two-way ANOVA test. (**d**) Tumor weights in mice treated with vehicle control, 5-FU, TH287, and MA−24. *, *p* < 0.05; ***, *p* < 0.001, ns, no significant difference, *t*-test. (**e**,**f**) Representative images and statistical results of lung metastases in mice treated with vehicle control, 5-FU, TH287, and MA−24. The small nodules on lung tissues indicate tumor lung metastasis (Black arrow), **, *p* < 0.01, *t*-test. (**g**) Representative images of double staining for Ki67 and nucleus. Scale bar, 200 μm. (**h**) Body weight changes in mice treated with vehicle control, 5-FU, TH287, and MA−24. ****, *p* < 0.0001, *t*-test.

To determine whether the in vitro inhibitory activity of target compounds can be translated into in vivo potency, we established a 4T1 breast cancer tumor model in Balb/c mice subcutaneously implanted with mammary carcinoma cells to assess the therapeutic efficacy of MA−24. Each group consisted of five tumor-bearing mice, which were, respectively, treated with5-fluorouracil (5-FU) (30 mg/kg), TH287 (200 mg/kg), MA−24 (60 mg/kg), or a solvent control. We used 5-FU and TH287 as positive controls, while a vehicle was used as a negative control. MA−24 significantly inhibited tumor growth compared to the vehicle (*p* < 0.0001) and positive control TH287 (tumor growth inhibition rate: 61.8% vs. 20.4%) (Figure 5a–d), and reduced lung metastasis (Figure 5e,f), indicating that MA−24, as an effective MTH1 inhibitor, possesses notable in vivo anti-tumor and anti-metastatic activity. Furthermore, we assessed the expression of Ki67, a molecular marker indicating cell proliferation in four groups. Our results show that 5-FU exhibits an inhibitory effect on tumor cell proliferation, and MA−24 also demonstrates a moderate reduction in Ki67 expression compared to the vehicle- or TH287-treated groups (Figure 5g). Additionally, comparing the changes in body weight among the groups, we found that 5-FU had the strongest tumor inhibitory effect in vivo (tumor growth inhibition rate: 81.9%), but also had the highest toxicity, leading to mouse mortality starting from the 16th day of treatment (Figure 5h). In contrast, the body weight of mice in the TH287 and MA−24 treatment groups showed no statistically significant differences compared to the control group, suggesting lower systemic toxicity and good safety profiles. These results indicate that, relative to the MA−24 can significantly inhibit tumor growth and lung metastasis in vivo, while exhibiting a lower systemic toxicity.

## 3. Discussion

In recent years, a diverse range of MTH1 inhibitors has been developed, including the structural optimization of existing inhibitors, screening for novel small molecule inhibitors from compound libraries, and extraction from natural products. The current state of breast cancer treatment indicates the need for the development of MTH1 inhibitors with improved activity. We identified MA−24 as an MTH1 inhibitor with anti-breast cancer effects through in vitro screening. We investigated the differential importance of MTH1 in normal and cancer cells and found that MA−24 outperformed conventional chemotherapeutic drugs in indiscriminately attacking both cancer and normal cells by specifically targeting cancer cells to exert its anticancer effects. Our study discovered the mechanisms of MA−24’s anti-MCF−7 cell effects at the DNA and protein levels. It significantly induced oxidative DNA damage in cells, inhibited cellular protective mechanisms by targeting MTH1, and activated apoptotic signals, ultimately leading to cell apoptosis. In an in vivo 4T1 tumor transplantation model, MA−24 exhibited significant inhibition of 4T1 tumor growth and metastasis. The considerable potential demonstrated by MA−24 in combating breast cancer provides theoretical and practical foundations for the development of new approaches to targeting MTH1 for breast cancer treatment. 

Currently, the combination of MTH1 inhibitors with other therapies provides potential strategies for cancer treatment. For example, MTH1 inhibition has been shown to alleviate immune suppression in experimental mesothelioma [28] and improve the efficacy of anti-PD-L1 immunotherapy, as well as enhance tumor sensitivity to radiotherapy [29]. Combination therapy with MTH1 inhibitors and reactive oxygen species enhancers can intensify oxidative damage, leading to increased cellular toxicity and the inhibition of tumor growth [30]. Additionally, TH287 has been shown to enhance tumor sensitivity to the anti-cancer drug NaAsO_2_ [31]. These studies demonstrate the broad application prospects of MTH1 inhibitors in cancer treatment.

Interestingly, comparing the in vitro and in vivo activity data of TH287 and MA−24, we found that, as can be seen in Figure 1 and Figure 2, TH287 exhibited much stronger enzymatic inhibition and cell proliferation inhibition activities compared to MA−24. However, in terms of tumor suppression in vivo, TH287 showed significantly weaker effects compared to MA−24. Through an analysis of the relevant literature, we speculate that this may be attributed to the instability of TH287 compound. In vitro studies have revealed that TH287 has a poorer stability compared to another MTH1 inhibitor [8], TH588. Furthermore, in another report, TH287 was found to be metabolized faster than TH588. Upon intraperitoneal injection at a dose of 5mg/kg, the blood drug concentration of TH287 was only 1 μΜ at 1 h, and the blood drug concentration at the time points of 2, 3, and 4 h dropped below the detection limit [32]. This indicates that TH287 is rapidly metabolized in vivo. Therefore, we hypothesize that MA−24 may have a longer half-life in vivo, and pharmacokinetic studies of MA−24 are necessary for further investigation.

The success of MTH1 inhibition in anticancer therapy sparked a research frenzy in this field. Nadia Gul et al. [33] have shown that microtubule proteins are the target of TH588 but not MTH1. Furthermore, Kettl. et al. [25] synthesized three distinct chemical series of MTH1 inhibitors and demonstrated the non-essential role of MTH1 in cancer cell survival, which challenged the research conclusions of Gad Helge and other research groups. [8] Interestingly, Warpman Berglund U et al. [34] later refuted the previously reported microtubule-targeting mechanism of TH588, demonstrating that TH588 has a distinct mode of action from microtubule-targeting drugs, such as paclitaxel and nocodazole, and emphasized the high selectivity of MTH1 inhibitors for MTH1 binding and inhibition. Subsequent studies once again affirm that MTH1 inhibition may provide a survival advantage to treated tumors by blocking DNA nicks caused by base excision repair and inducing p53 [35]. Warpman Berglund U further demonstrated that the MTH1 inhibitor TH1579 is an anticancer agent for acute myeloid leukemia via inducing oxidative DNA damage and mitotic arrest [36]. This study aimed to report the therapeutic effect of novel MTH1 inhibitors MA−24 in breast cancer treatment. Additionally, we elucidated the necessity of MTH1 for the survival of breast cancer cells. We noticed that MTH1 overexpression rescued the damage caused by the MA−24 in MCF−7 cells, while the MTH1 knockdown group still showed susceptibility to MA−24 in Figure 3e, f. There are two possible reasons for this observation: a, in the MTH1-silenced cells, MTH1 expression is reduced but not completely abolished, and the remaining MTH1 is affected by the inhibition of MA−24, leading to changes in cell viability. And b, MA−24 may have other targets apart from MTH1 that collectively impede the survival of MCF−7 cells. The off-target effects of MA−24 cannot be completely ruled out, which drives us to search for potential targets other than MTH1. Additionally, in MCF−7 cells, it has been reported that the inhibition of MTH1 increases the proportion of CD44^+^ CD24^−/Low^ cell subpopulations, suggesting that MTH1 inhibition enhances the number of breast cancer stem cells [37]. This poses another challenge for MTH1 inhibitors in the process of drug translation. It highlights the complexity of the mechanism of action of MTH1 inhibitors. Although we cannot exclude the possibility of off-target effects, our current research has not identified any other target that is more compelling than MTH1, which prompts us to further investigate this matter. In conclusion, this study provides a theoretical and practical foundation for the development of new approaches targeting MTH1 for breast cancer treatment.

## 4. Materials and Methods

### 4.1. Compounds

2,4,6−Triaminopyrimidine derivatives MA−19–MA−24 and 2,4,6−triamino−1,3,5−triazine derivatives MB−16–MA−17 were kindly provided by Professor Yisheng Lai (China Pharmaceutical University). We purchase 5-FU and TH287 hydrochloride from Aladdin (Shanghai, China) and Dalian Meilun Biotech Co., Ltd. (Dalian, China), respectively. The structure and purity of the compounds were checked using MS and HNMR.

### 4.2. IC_50_ Determination

MTH1, inorganic pyrophosphatase, and dGTP (Thermo Fisher Scientific, Waltham, MA, USA) were diluted in assay buffer containing 100 mM Tris-acetate at pH 8.0, 40 mM NaCl, 10 mM Mg-acetate, 0.005% Tween 20, and 1 mM DTT. Briefly, serial dilutions of compounds were dissolved in assay buffer; meanwhile, MTH1 was diluted to 4 μg/mL, and then these two solutions were mixed and filled with buffer to 186 µL. After 15 min incubation at 25 °C, 10 µL dGTP (final concentration 100 mM) and 4 µL inorganic pyrophosphatase (final concentration 0.2 U/mL) were added to start the reaction. The reaction mixture was incubated with shaking for 45 min at 25°C. Finally, 50 µL of malachite green assay reagent was added and incubated with shaking for 15 min at 25 °C. The absorbance of the assay plate was read at 630 nm using a Multiskan GO Microplate Spectrophotometer (Thermo Fisher Scientific, Waltham, MA, USA) and IC_50_ values were calculated using GraphPad Prism 9.5.1 Software.

### 4.3. Cell Lines and Cell Culture

Human breast cancer cell lines (MCF−7: ER+, PR+, HER2−, EGFR+, MB−MDA−MB−231: TNBC, EGFR+), a murine mammary cancer cell line (4T1, TNBC), immortalized cell lines, and HEK-293F, CHO, and HUVEC cells were obtained from ATCC. All cells were cultured in suitable medium supplemented with 10% fetal bovine serum (FBS; Zhejiang Tianhang Biotechnology Co., Ltd., Hangzhou, China), 100 U/mL penicillin, and 100 μg/mL streptomycin (Invitrogen, Carlsbad, CA, USA) at 37 °C in 5% CO_2_. MCF−7, MB−MDA−MB−231, HEK-293F, and HUVEC were grown in Dulbecco’s modified Eagle’s medium (DMEM; Invitrogen, Carlsbad, CA, USA), 4T1 were cultivated in Roswell Park Memorial Institute (RPMI)-1640 medium (Invitrogen, Carlsbad, CA, USA), and CHO cells were grown in Ham’s F-12 Nutrient Mix (Invitrogen, Carlsbad, CA, USA).

### 4.4. Cell Viability Assays

Viability of the cells was measured using MTT assay (Aladdin, Shanghai, China), where cells were seeded into 96-well plates at densities of 4000 (MCF−7, 4T1, HUVEC and CHO) and 8000 (MB−MDA−MB−231 and HEK-293F) cells per well and grown for 24 h. Subsequently, cells were treated with compound or vehicle (maximum 0.3% DMSO). After 48 h, cells were treated with MTT; the absorbance of the assay plate was read at 490 nm using a Multiskan GO Microplate Spectrophotometer (Thermo Fisher Scientific, Waltham, MA, USA). Each experiment was performed in triplicate and IC50 values were calculated using GraphPad Prism 9.5.1 Software Inc., nonlinear curve fit with variable slope (four parameters).

### 4.5. Annexin V/PI Apoptosis Assay

3 × 10^6^ MCF−7 cells were seeded into 6-well plates and cultured at 37 °C with 5% CO_2_ for 24 h. Then, cells were exposed to the tested compound or vehicle (maximum 0.3% DMSO) and, after 48 h, were treated with Annexin V-FITC/PI Apoptosis Detection Kit per the manufacturer’s instructions (Cwbio, Nanjing, China). Cells were analyzed on a MACSQuant™ flow cytometer (Miltenyi Biotec, Cologne, Germany) using FlowJo V10.6.2 software. 

### 4.6. Comet Assay

MCF−7 cells (300,000 cells) were plated in 6-well plates and incubated for 24 h at 37 °C and 5% CO_2_. Subsequently, cells were treated with a compound (10 μM MA−24 and TH287) or vehicle (0.1% DMSO) for 2 days or with 150 μM for 10 min on ice. Cells were harvested as above. After washing twice with PBS, cells were re-suspended in PBS. Then, the 30 μL cell suspension was mixed with 150 µL 1.2% low-melting agarose that was maintained at 37 °C. The mixture was layered onto pre-warmed (37 °C) agarose-coated glass slides. The slides were kept on ice for 10 min and incubated in lysis buffer (10 mM Tris pH 7.7, 2.5 M NaCl, 0.1 M EDTA, 1% DMSO, and 1% Triton X-100) at 4 °C overnight in the dark. Slides were washed twice with enzyme reaction buffer (40 mM HEPES pH 8.0, 0.1 M KCl, 0.5 mM EDTA, and 0.2 mg/mL BSA) and incubated with Fpg buffer alone (8 U/mL, New England Biolabs, Shanghai, China) at 37 °C for 45 min. Slides were washed once with enzyme reaction buffer and incubated in alkaline electrophoresis buffer (50 mM NaOH, 1 mM EDTA, and 1% DMSO) for 20 min. Electrophoresis was run at 300 mA, 25 V for 30 min in electrophoresis buffer. Slides were washed in neutralization buffer (0.4 M Tris-HCl pH 7.0) and stained with 3 μg/mL PI (Sigma Aldrich, Taufkirchen, Germany). Images were acquired with a fluorescence microscope (Olympus Corporation, Tokyo, Japan) and quantified using Comet Score software, Version 1.5. At least 100 comets per sample were analyzed. Tail moment is calculated as the percentage of DNA in the tail multiplied by the tail length.

### 4.7. Western Blot Analysis

Total protein was obtained using RIPA lysis buffer (Beyotime Biotechnology, Shanghai, China) supplemented with phenylmethanesulfonyl fluoride (Beyotime Biotechnology, Shanghai, China), separated by SDS-PAGE, and transferred to the PVDF membrane by electroblotting (Bio-Rad, Hercules, CA, USA) followed by blocking with 5% milk in TBST. MTH1 antibody (Cat.No.ab200832, Abcam, Eugene, OR, USA), β-actin antibody (Cat.No.ab8227; Abcam, Eugene, OR, USA), ccaspae-3 antibody (Cat.No.14220, CST, Boston, USA), cleaved caspase-3 antibody (Asp175) (Cat.No.9664, CST, Boston, MA, USA), cleaved PARP antibody (Asp214) (Cat.No.95696, CST, Boston, MA, USA), p38 (Cat.No.9212, CST, Boston, MA, USA), and phospho-p38 MAPK (Thr180/Tyr182) (Cat.No.9211, CST, Boston, MA, USA) were used at a dilution of 1:1000–1:5000. Upon addition of primary and HRP-conjugated secondary antibodies, blots were detected using an ECL substrate (Thermo Fisher Scientific, Waltham, MA, USA) and the Tanon 5200 Chemiluminescent Imaging System (Tanon, Shanghai, China). Band intensities were quantified using densitometry Image J 1.49 version software.

### 4.8. Target Engagement Assay

MCF−7 cells cultured in 6-well plates for 24 h were exposed to vehicle (0.5% DMSO) or 50 μM TH287 or MA−24 for 4.5 h. After trypsinization cells were detached, spun down and resuspended in PBS. Eight equational cells were heated for 3 min from 42 to 75 °C, and then lysed using RIPA lysis buffer. Western blot analysis was used to analyze the amounts of stabilized MTH1 using the MTH1 antibody. 

### 4.9. RNA Interference 

MCF−7 cells cultured in 6-well plates to 70% confluency were transfected with 100 nM siRNA oligos (GenePharma, Shanghai, China) and siRNA transfection reagent (Invitrogen, Waltham, MA, USA) according to the manufacturer’s protocol. After 48 h, knockdown was verified by Western blot. Target sequences were as follows: MTH1 siRNA#1 5′−CCUGCUUCAGAAGAAGAAATT−3′, MTH1 siRNA#2 5′−AGGAGAGACCAUCGAGGAUTT−3′, MTH1 siRNA#3 5′−GGUUCCAGCUGGAUCAGAUTT-3′, control scrambled siRNA 5′−UUCUCC GAACGUGUCACGUTT−3′.

### 4.10. MTH1 Overexpression Studies 

The mth1 cDNA was obtained by PCR amplification and was inserted into pDsRed1-N1 with EcoRI and XhoI restriction sites. The pDsRed1-N1 MTH1 construct was identified by sequencing. MCF−7 cells were transfected with pDsRed1-N1 vector or pDsRed1-N1 MTH1 construct using Lipofectamine^®^ 2000 Reagent (Invitrogen, Waltham, MA, USA) according to the manufacturer’s instruction. After 48h, the overexpression of MTH1 was verified by Western blot.

### 4.11. Animals and In Vivo Efficacy Studies

All animal experiments were carried out in accordance with a protocol approved by the Animal Ethics Committee of the China Pharmaceutical University. Female BALB/C mice were given food and water ad libitum. Approximately 7-week-old mice were injected subcutaneously with 2 × 10^5^ 4T1 cells in the left flank. When the mean tumor size reached 30~50 mm^3^, vehicle (composed with 1% DMSO, 10% ethanol, 10% Chremophor EL, and 10% Tween-80, diluted with PBS), MTH1 inhibitor (MA−24 at 60 mg/kg or TH287 at 200 mg/kg), or 5-FU (30 mg/kg) was administered intraperitoneally every three days. Tumor size and body weights were measured twice weekly starting on the first day of treatment. Moreover, to minimize animal suffering, euthanasia by cervical dislocation was performed when the maximum diameter of the tumors exceeded 1 cm.

### 4.12. Molecular Docking Method

Molecular docking was completed with Glide 5.5 implemented in Schrödinger2009. The crystal structure (PDB ID: 4N1T) of MTH1 in complex with TH287 was obtained from the Protein Data Bank (http://www.rcsb.org/, accessed on 8 December 2022). The crystal structure was prepared with the Protein Preparation Wizard workflow. All of the water molecules were removed. The grid file for molecular docking was generated based on the binding site, which was defined by a box centered on the centroid of the crystal ligand and with a similar size to it. Compounds were prepared with LigPrep and docked using the Glide extra-precision (XP) mode. Default settings were used for all of the other parameters.

### 4.13. Statistical Analysis

Data are presented as mean ± standard deviation (SD). The Student’s *t*-test or two-way ANOVA test was used for determining statistical significance between groups (*, *p* < 0.05; **, *p* < 0.01; ***, *p* < 0.001; ns, no significant difference). All experiments were repeated three times or more.

## Figures and Tables

**Table 1:** Compounds MA−19–MA−25 and MB−16–MB−17 were evaluated for their inhibitory effects on the proliferation of MCF−7 cells at a concentration of 200 μM. The results demonstrated that MA−24 and MA−25 exhibited a close to 100% inhibition rate at the concentration of 200 μM.

Compounds (Concentration: 200 μM)	Inhibition (%) (Mean ± SD)
MA−19	37.8 ± 8.8
MA−20	44.2 ± 8.4
MA−21	28.1 ± 1.5
MA−22	30.3 ± 4.1
MA−23	11.8 ± 4.5
MA−24	99.6 ± 0.4
MA−25	100.0 ± 0.1
MB−16	52.1 ± 2.9
MB−17	27.3 ± 4.2

## Data Availability

All original data can be obtained by contacting the corresponding author, Liu Yu (liuyu@cpu.edu.cn).

## Supplementary Materials

The following supporting information can be downloaded at: https://www.mdpi.com/article/10.3390/ph17030291/s1, Figure S1: Chemical structures of compounds MA−19–MA−23, MB−16, and MB−17.
