# Supplementary figures and images for: Targeting MutT Homolog 1 (MTH1) for Breast Cancer Suppression by Using a Novel MTH1 Inhibitor MA−24 with Tumor-Selective Toxicity
Source: Pharmaceuticals (Basel). 2024 Feb 23;17(3):291. doi: 10.3390/ph17030291 (PMC10974945; doi:10.3390/ph17030291)

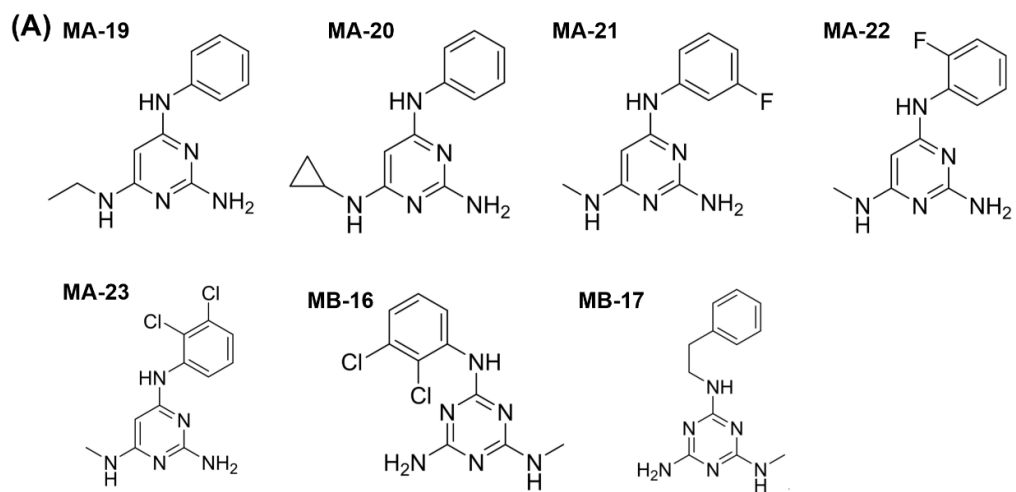

**Figure S1.** Chemical structures of compounds MA-19 to MA-23, MB-16, and MB-17.

Supplement: Supplementary file 1 [file pharmaceuticals-17-00291-s001.zip › pharmaceuticals-2858417-supplementary.pdf]
